# Supplementary material for: NFAT5/TonEBP Limits Pulmonary Vascular Resistance in the Hypoxic Lung by Controlling Mitochondrial Reactive Oxygen Species Generation in Arterial Smooth Muscle Cells
Source: Cells. 2021 Nov 24;10(12):3293. doi: 10.3390/cells10123293 (PMC8699676; doi:10.3390/cells10123293)
Supplement: Supplementary file 1 [file cells-10-03293-s001.zip › cells-1448758-supplementary.pdf]

Supplement 1

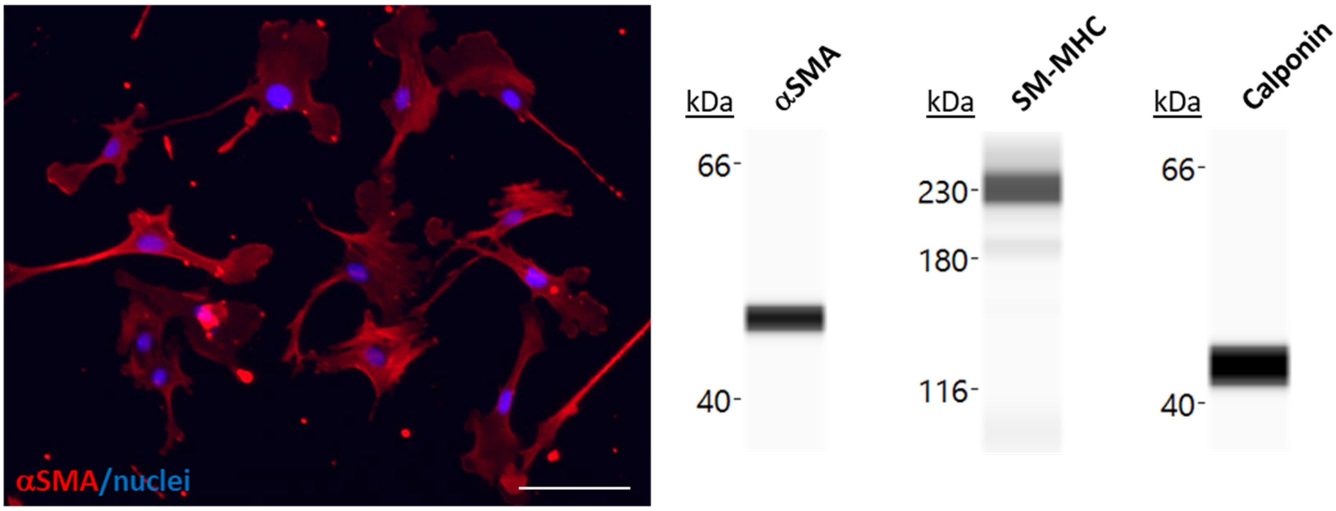

Murine pulmonary artery smooth muscle cells (mpaSMCs) were cultured and SMC markers (αSMA, SM-MHC, calponin) were detected by immunofluorescence-based techniques (αSMA - red fluorescence, top image, scale bar: 100 µm) or immunodetection in lysates after capillary electrophoresis (lower panel).

Supplement 2

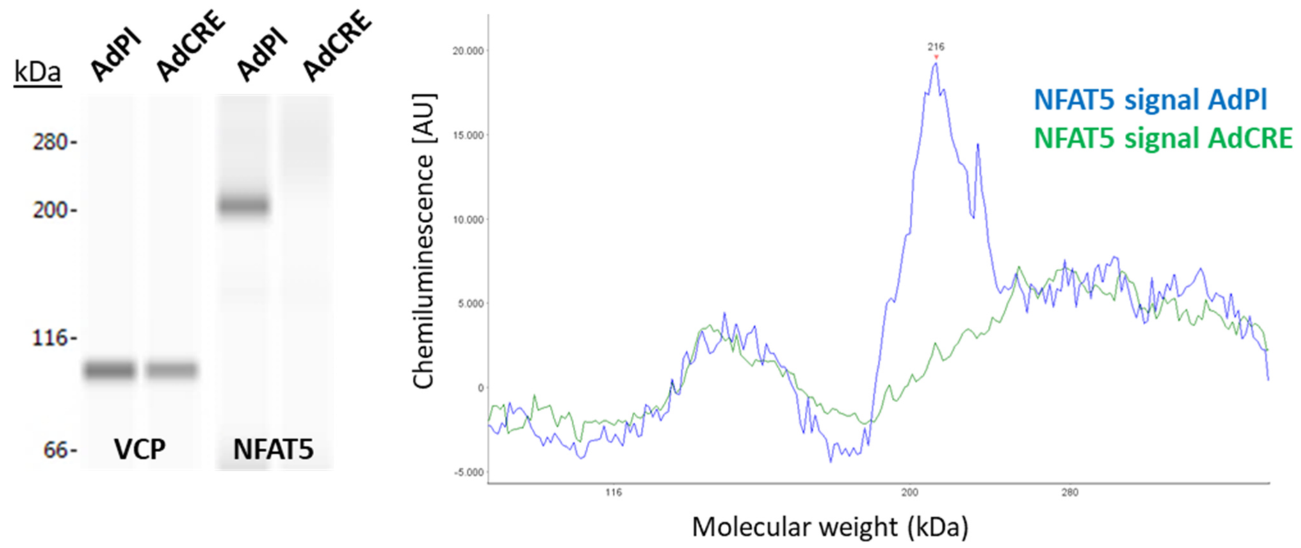

Capillary electrophoresis-based immunodetection of NFAT5 in lysates of maoSMCs treated with AdCRE adenovirus to genetically ablate *Nfat5* (*Nfat5*$^{-/-}$) or AdPl adenovirus as a control (*Nfat5*$^{fl/fl}$). VCP (valosin containing protein) served as loading reference. The right panel shows the corresponding signal peaks detected by the analysis software.

Supplement 3

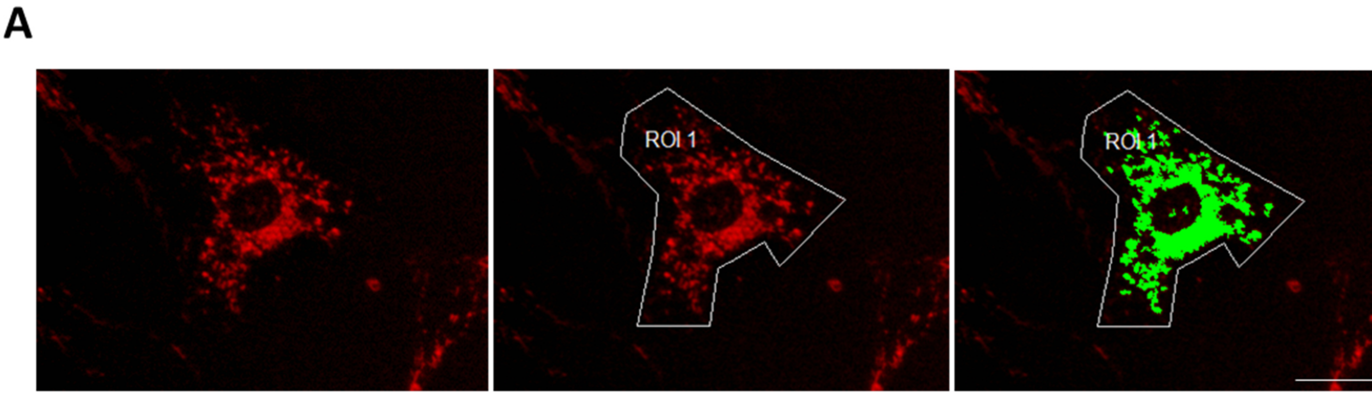

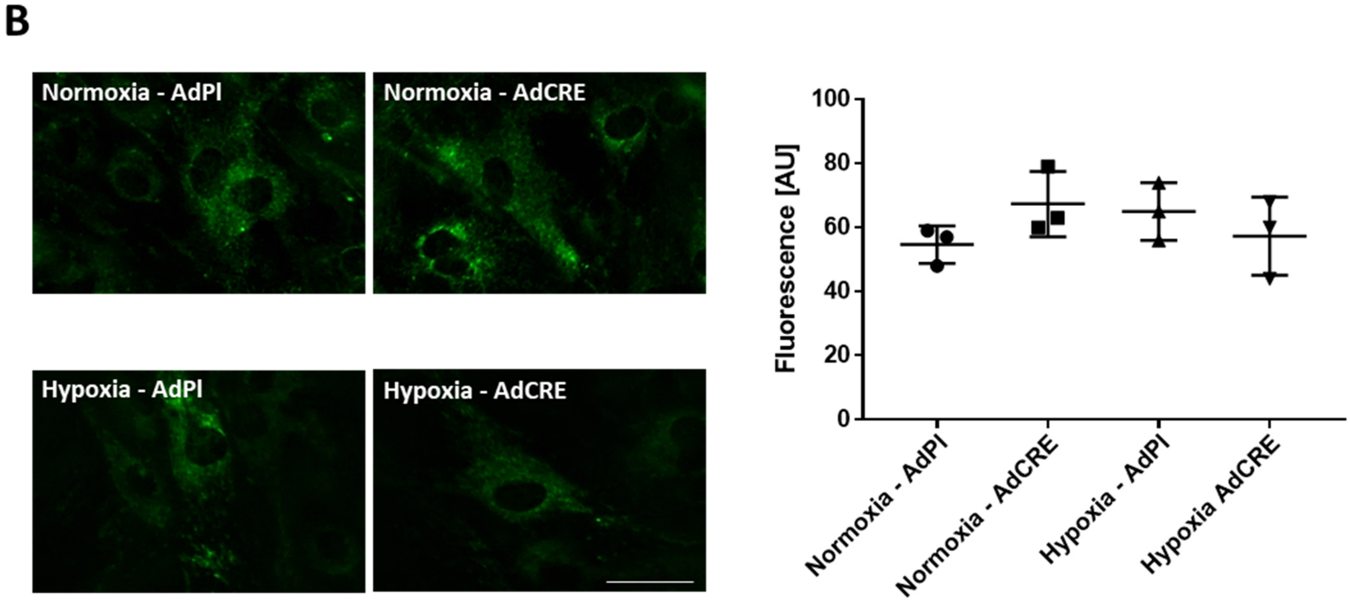

(A) Immunofluorescence-based mitochondrial ROS detection in maoSMCs (red fluorescence) and its software-supported evaluation in a region of interest (ROI, green color, right image, scale bar: 50 µm). **(B)** Detection of mitochondria by the fluorophore MitoTracker™ Green FM in maoSMCs treated with AdCRE adenovirus to knockout *Nfat5* or AdPl adenovirus as a control and exposed to normoxia/hypoxia for 24 h. The graph summarizes the data of one experiment performed in triplicate. No significant changes were observed (scale bar: 50 µm).

Supplement 4

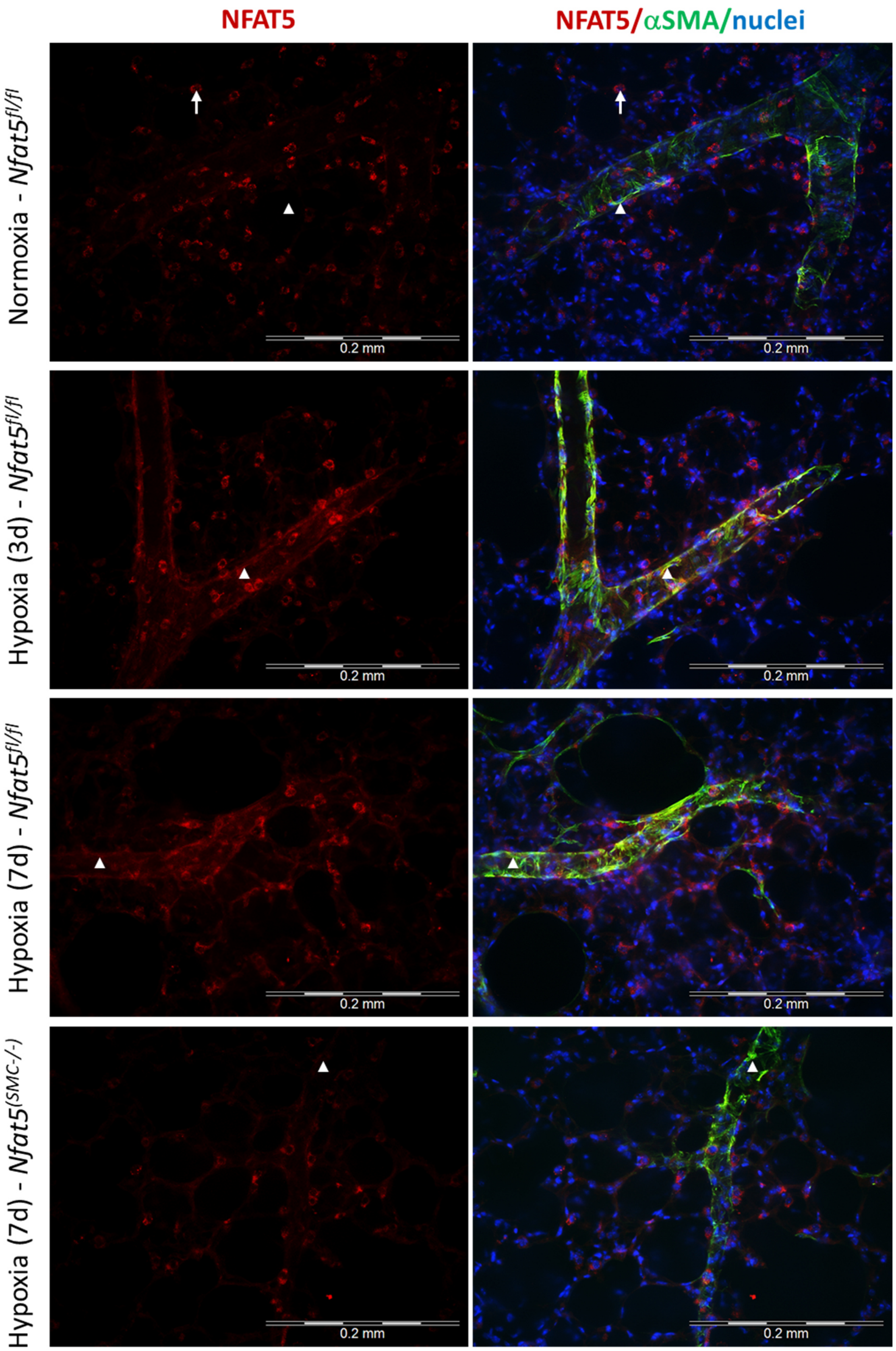

Vibratome sections of lungs from hypoxia/normoxia-exposed *Nfat5*$^{fl/fl}$ and *Nfat5*$^{(SMC)-/-}$ mice were processed to detect αSMA (green) and NFAT5 (red) by immunofluorescence-based techniques (directly labelled antibodies were utilized only) and image stacks were generated by confocal microscopy (scale bars: 200 µm, note that NFAT5 localized in the nuclei cannot be detected in formalin-fixed vibratome sections). In normoxic lungs, NFAT5 was preferentially detectable in immune cells (arrows) but not in vascular smooth muscle cells (arrowheads). NFAT5-associated IF was detectable in αSMA-positive cells in hypoxia-exposed *Nfat5*$^{fl/fl}$ but not *Nfat5*$^{(SMC)-/-}$ lungs.

Supplement 5

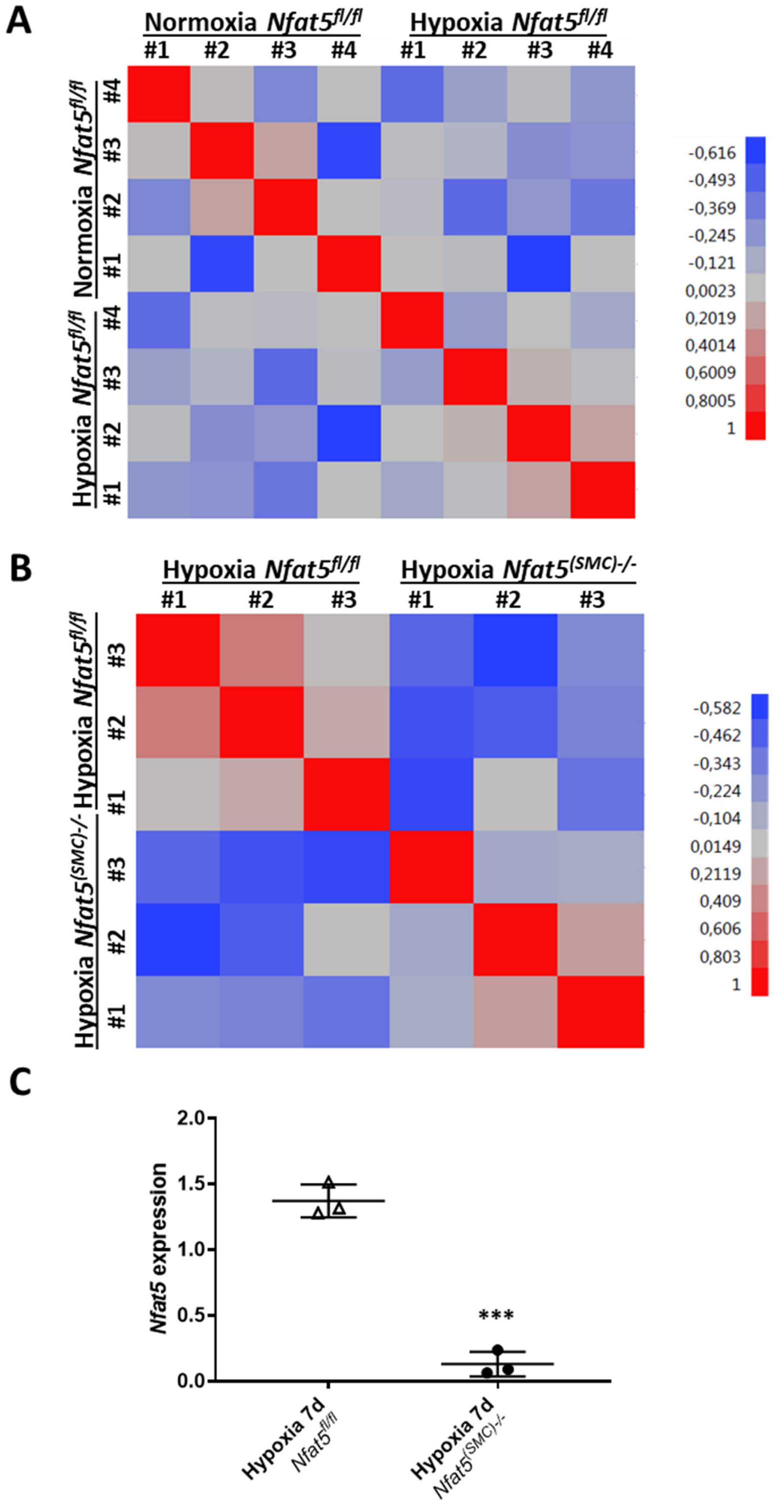

(A,B) Principal component analyses of RNA samples of lungs from *Nfat5*$^{fl/fl}$ mice exposed to normoxia/hypoxia for 7 d (A, n=4) and from *Nfat5*$^{(SMC)-/-}$ and *Nfat5*$^{fl/fl}$ mice exposed to hypoxia for 7 d (B, n=3). The hierarchical clustering analysis indicated a homogenous correlation of the clustering of samples from individual experimental groups. **(C)** qPCR-based comparison of the expression of *Nfat5* (transcriptional target of NFAT5) in lungs from *Nfat5*$^{(SMC)-/-}$ and *Nfat5*$^{fl/fl}$ mice exposed to hypoxia for 7 d (***p<0.001 vs. *Nfat5*$^{fl/fl}$ mice, n=3).

Supplement 6

Gene name	Category	FR (log2) - *Nfat5*$^{fl/fl}$: Hyp. vs. Norm.	p-value	FR (log2) - Hyp.: *Nfat5*$^{(SMC)-/-}$ vs. *Nfat5*$^{fl/fl}$	p-value
Nfat5	Transcription factor	n.s.	>0.05	-1.06	**<0.01**
Pdgfb	SMC biology	0.71	**<0.01**	n.s.	>0.05
Pdgfrb	SMC biology	n.s.	>0.05	n.s.	>0.05
Myh2	SMC biology	n.s.	>0.05	n.s.	>0.05
Myh11 (SMMHC)	SMC biology	n.s.	>0.05	n.s.	>0.05
Myl4	SMC biology	-0.64	**<0.01**	n.s.	>0.05
Myl7	SMC biology	-1.05	**<0.001**	n.s.	>0.05
Vim	SMC biology	0.45	**<0.05**	n.s.	>0.05
Tagln (SM22)	SMC biology	0.62	**<0.05**	n.s.	>0.05
Acta2 (aSMA)	SMC biology	0.71	**<0.05**	n.s.	>0.05
Cacna1c	SMC biology	0.84	**<0.01**	-0.77	**<0.01**
Ednra	SMC biology	n.s.	>0.05	-0.44	**<0.01**
Plcb1	SMC biology	n.s.	>0.05	-0.70	**<0.01**
Prkg1	SMC biology	-0.71	**<0.05**	-0.71	**<0.05**
Rock1	SMC biology	n.s.	>0.05	-0.50	**<0.05**
Rock2	SMC biology	n.s.	>0.05	-0.69	**<0.05**

Expression of individual genes associated with the SMC phenotype in lungs of *Nfat5*$^{fl/fl}$ and *Nfat5*$^{(SMC)-/-}$ mice exposed to normoxia/hypoxia for 7 days. The left panel lists significantly regulated genes (downregulated: green, upregulated: red, FR – fold regulation (log2-values)) when comparing hypoxia (7d)/*Nfat5*$^{fl/fl}$ and the corresponding normoxia/*Nfat5*$^{fl/fl}$ group (left panel, n=4, n.s. – not significant). The right panel shows the change in gene expression comparing hypoxia-exposed (7 d) *Nfat5*$^{(SMC)-/-}$ and *Nfat5*$^{fl/fl}$ mice (n=3).

Supplement 7

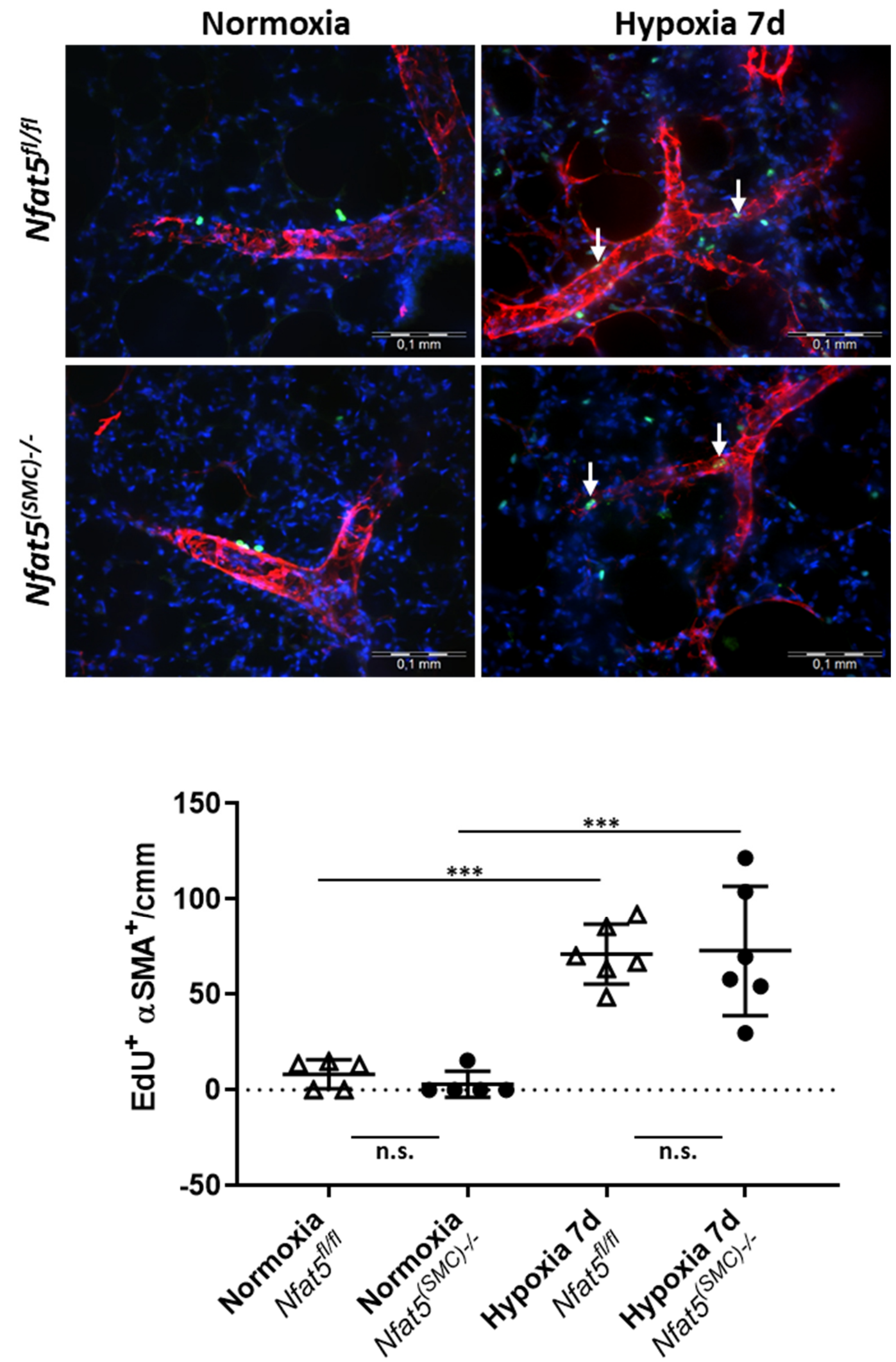

Vibratome sections of lungs from hypoxia/normoxia-exposed *Nfat5*$^{(SMC)-/-}$ and *Nfat5*$^{fl/fl}$ mice were processed to detect EdU (green fluorescence, labels cells with DNA synthesis during the last 2 hours of the experiment) and the SMC marker αSMA (red fluorescence) by immunofluorescence-based techniques (scale bars: 100 µm). Images stacks of confocal images were morphometrically evaluated to determine the number of EdU^{+}/αSMA^{+} cells (arrows) per cubic mm in arterial segments with a diameter <30 µm (n.s.- not significant, ***p<0.001 as indicated, n=5-6).

Supplement 8

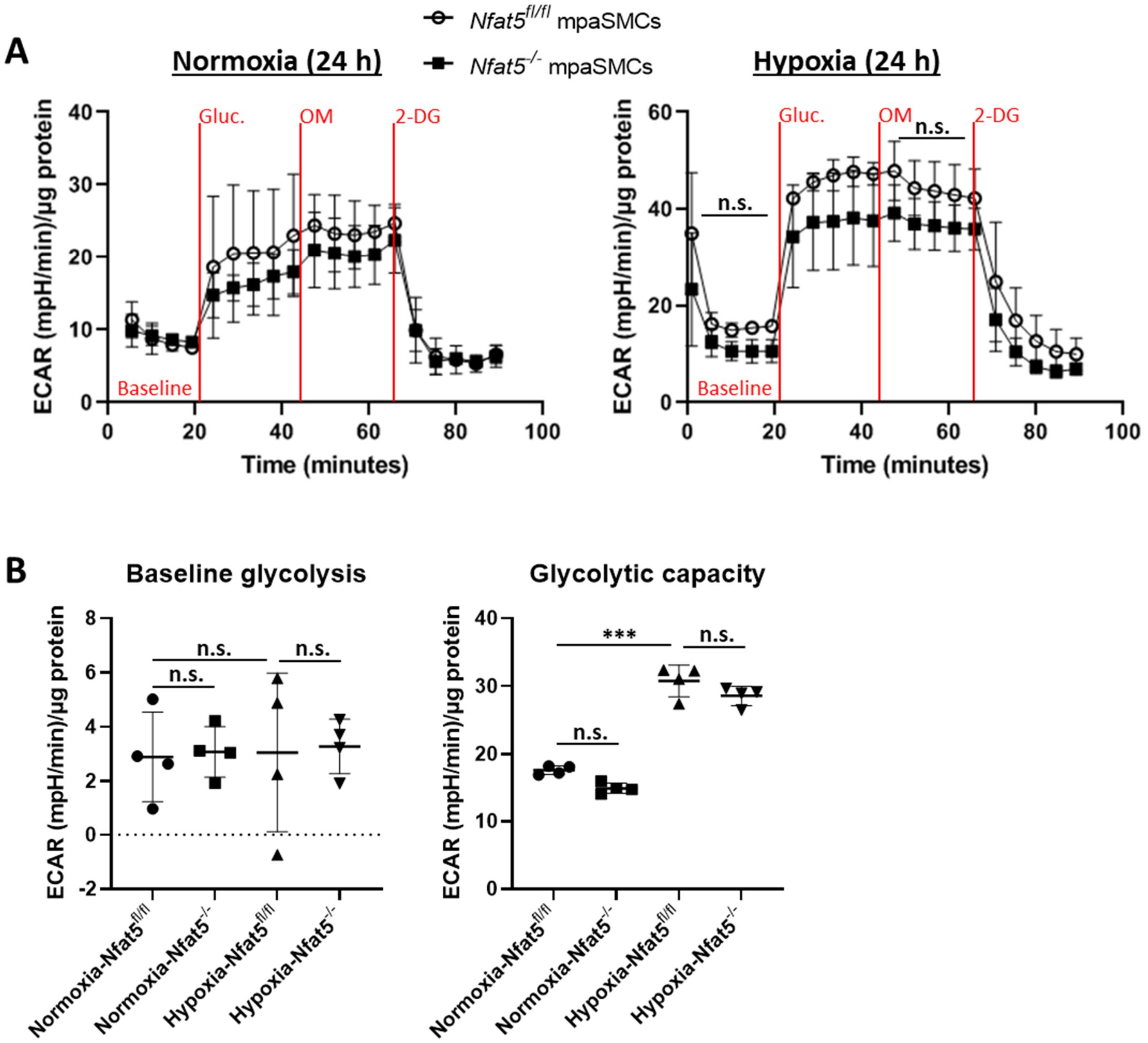

Glycolysis was assessed by analysis of the extracellular acidification rate (ECAR) using a Seahorse XF analyzer. mpaSMCs were treated with adeno-associated virus to overexpress CRE recombinase (*Nfat5*$^{-/-}$) or GFP (*Nfat5*$^{fl/fl}$) and exposed to normoxia/hypoxia for 24 h prior to analysis. **(A)** ECAR was determined while treating the cells with glucose (Gluc.), oligomycin (OM) and 2-desoxy-D-glucose (2-DG) to analyze **(B)** baseline glycolysis and glycolytic capacity. Three replicates were performed for each sample (***p<0.001 as indicated). ECAR was normalized to the total cellular protein per sample.

Supplement 9

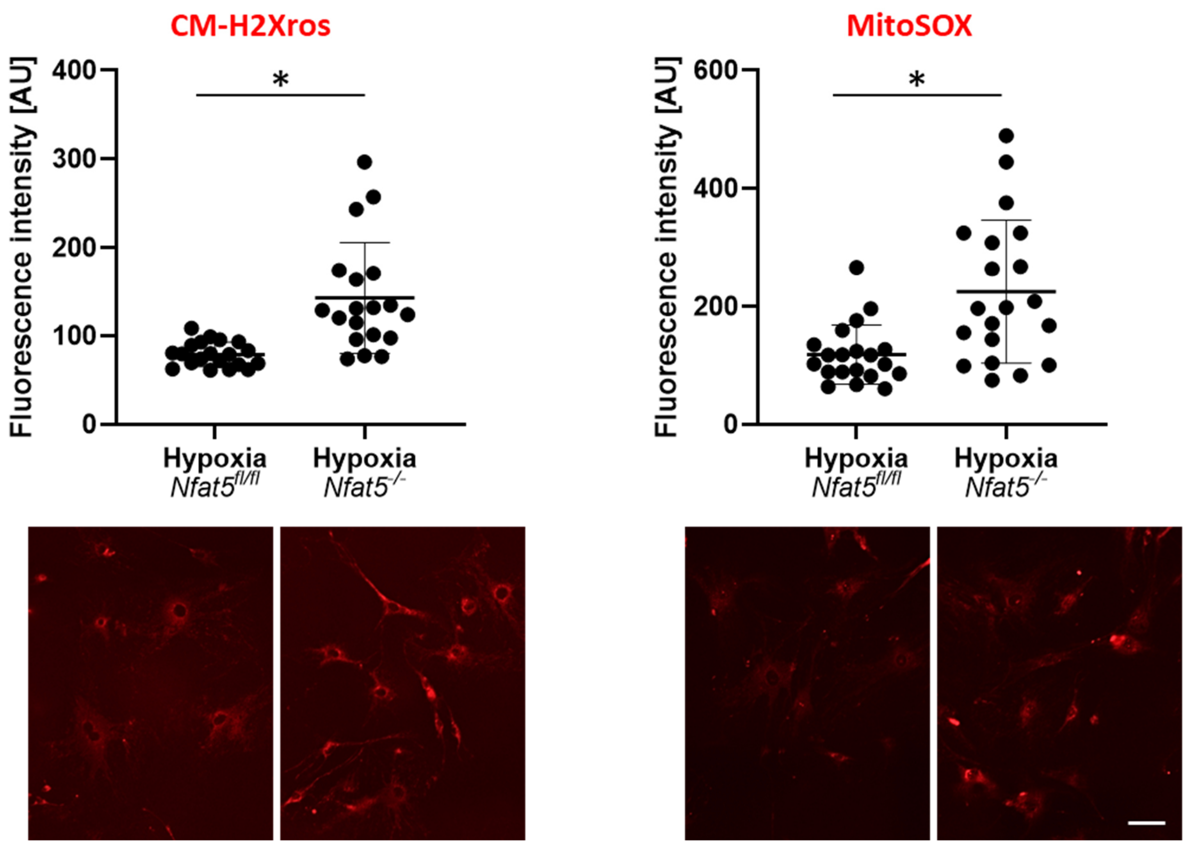

Mitochondrial ROS production was assessed by determining the fluorescence intensity of ROS-sensitive mitochondrion-selective probes (MitoTracker™ Red CM-H2Xros and MitoSOX Red) in mpaSMCs from *Nfat5*$^{fl/fl}$ mice, treated with control (*Nfat5*$^{fl/fl}$) and Cre recombinase-transducing (*Nfat5*$^{-/-}$) adenoviral vectors and exposed to hypoxia for 24 h (*p<0.05 as indicated, the results of 19-20 randomly selected regions of interest (ROI) from one experiment are shown, scale bar: 50 µm).

Supplement 10

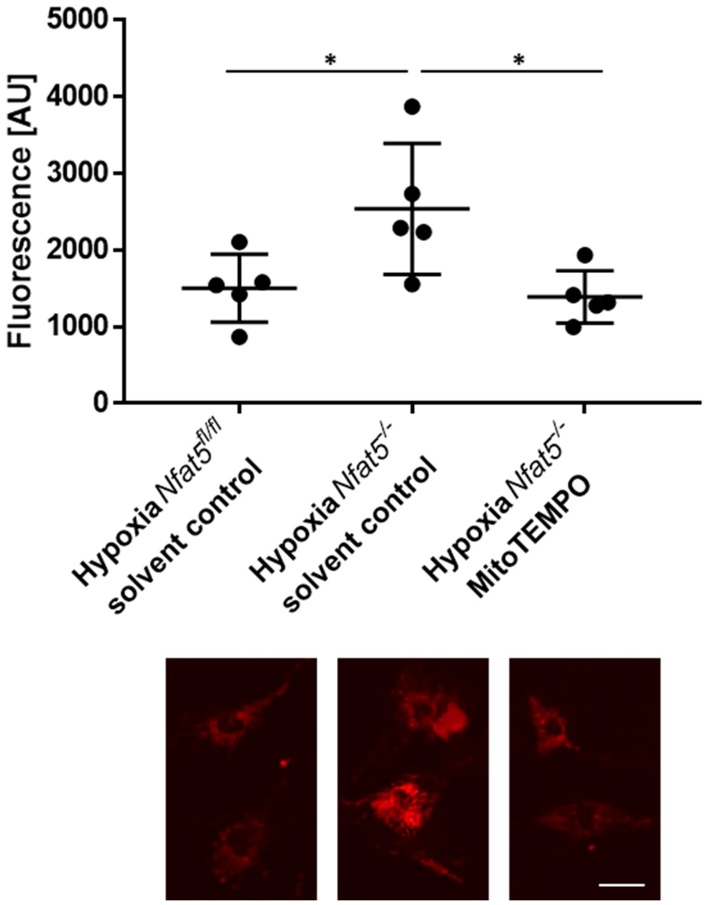

Mitochondrial ROS production was assessed by recording the fluorescence intensity of a ROS-sensitive mitochondrion-selective probe (MitoTracker™ Red CM-H2Xros) in mpaSMCs, treated with adenoviral vectors (AdCRE) to genetically ablate *Nfat5* (*Nfat5*$^{-/-}$) or AdPl vectors as control (*Nfat5*$^{fl/fl}$) and exposed to hypoxia for 24 h. Hypoxia-exposed (24 h) *Nfat5*$^{-/-}$ mpaSMCs were treated with the mitochondrion-specific ROS scavenger MitoTEMPO (20 µM) for 1 h before fluorescence recording (*p<0.05 as indicated, the results of one experiment performed in pentaplicate are shown, scale bar: 50 µm).

Supplement 11

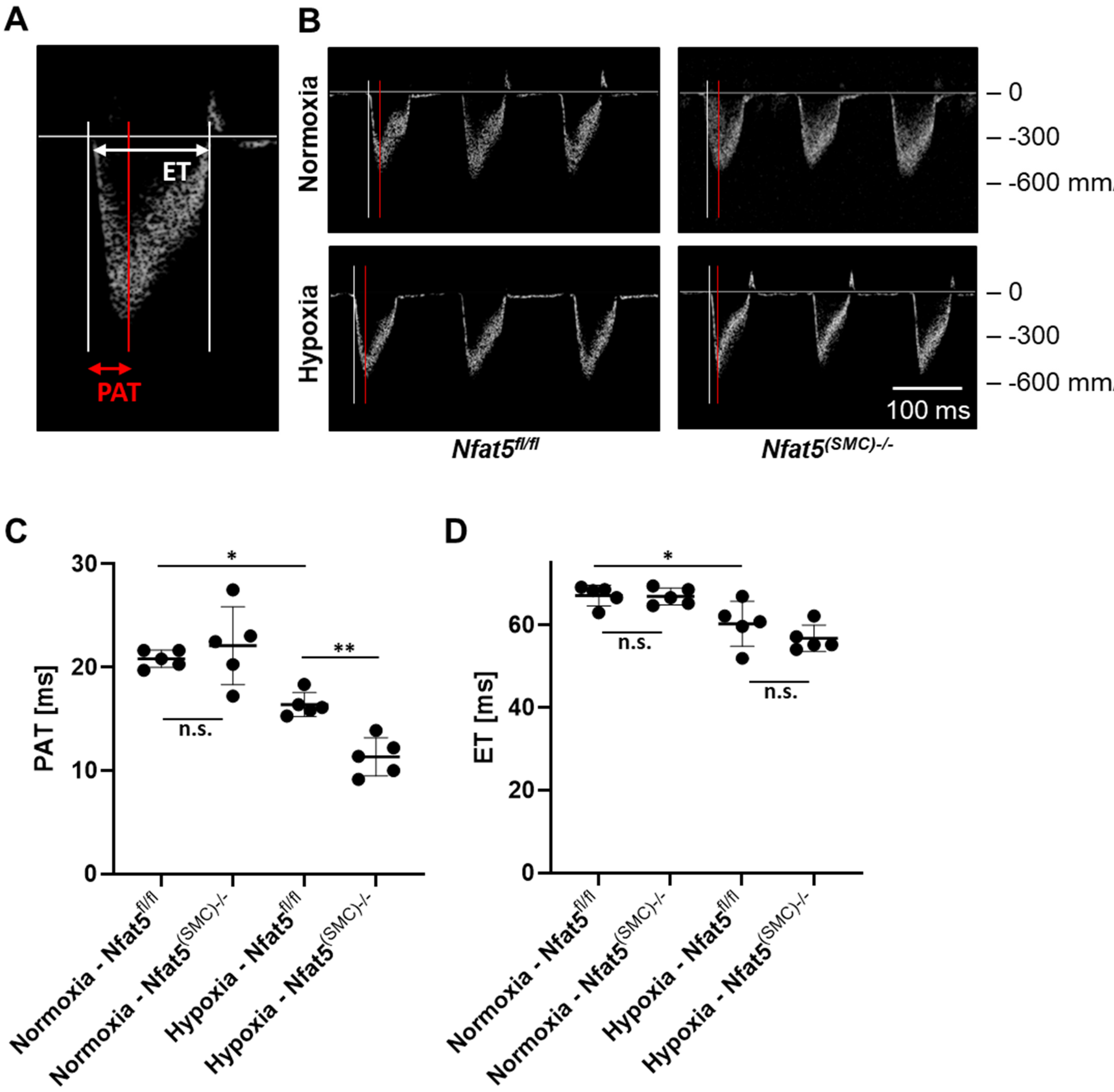

(A) Echocardiographic analysis of right venticular pulmonary acceleration time (PAT) and ejection time (ET). **(B)** Representative (Doppler) echocardiographic recordings of the pulmonary artery ejection in *Nfat5*$^{fl/fl}$ and *Nfat5*$^{(SMC)-/-}$ mice exposed to normoxia/hypoxia for 21 d. **(C and D)** Graphs summarizing individual PAT and ET values from mice (*p<0.05, **p<0.01 as indicated, n.s. – not significant, n=5). Data is shown as mean ±SD.

Supplement 12

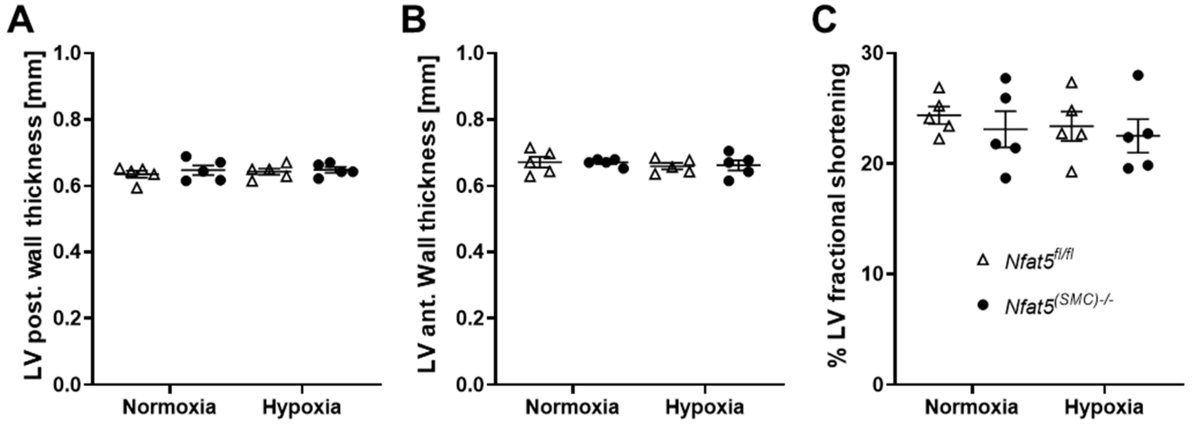

(A-C) Echocardiographic analysis of left venticular (LV) structural and functional parameters of *Nfat5*$^{fl/fl}$ and *Nfat5*$^{(SMC)-/-}$ mice exposed to normoxia/hypoxia for 21 d. No significant differences were observed (n=5). Data is shown as mean ±SEM.

Supplement 13

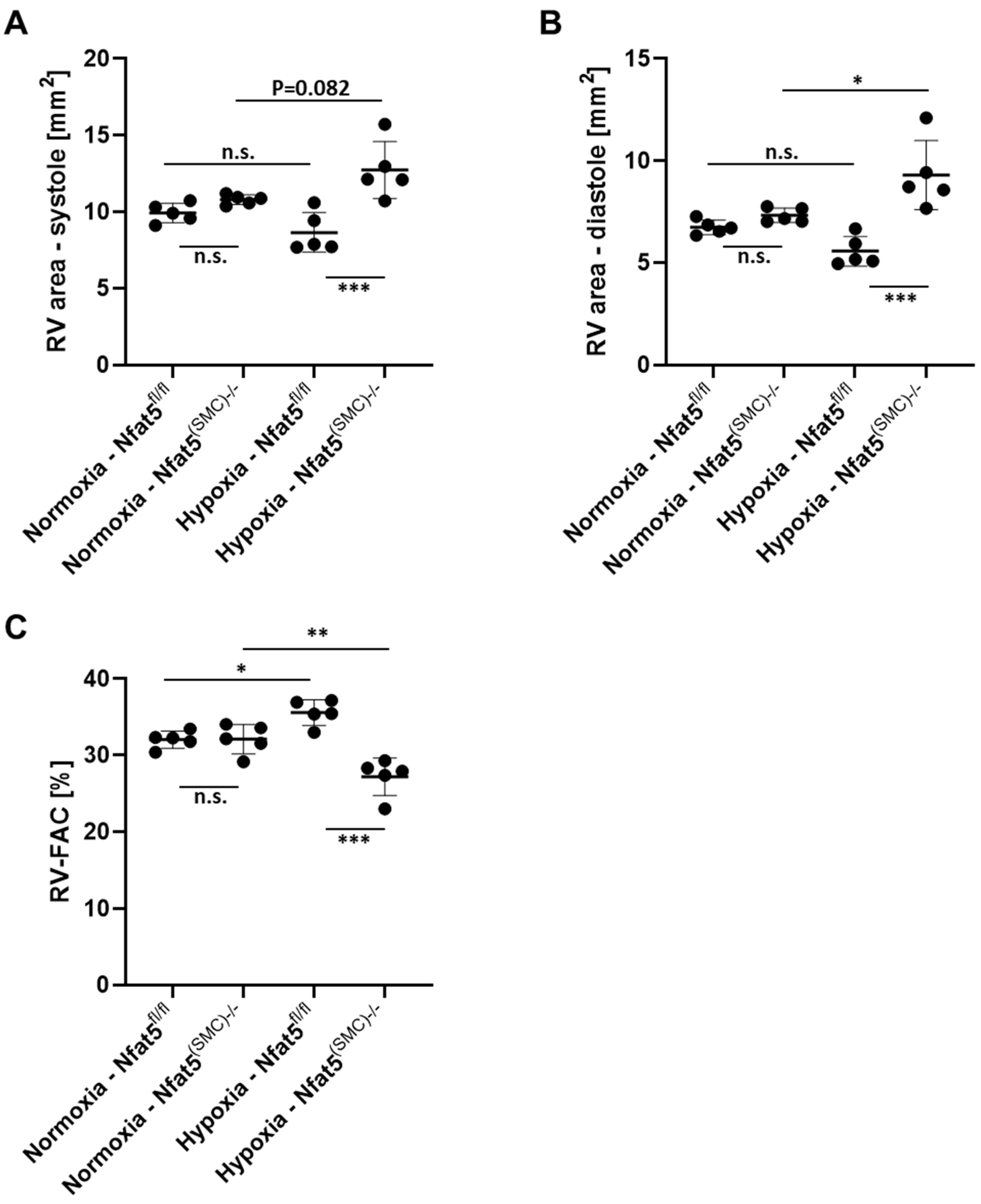

(A-C) Echocardiographic data of right venticular (RV) area during systole (A) and diastole (B) as well as (C) RV fractional area change (FAC) in *Nfat5*$^{fl/fl}$ and *Nfat5*$^{(SMC)-/-}$ mice exposed to normoxia/hypoxia for 21 d (*p<0.05,**p<0.01, ***p<0.001 as indicated, n.s. – not significant, n=5). Data is shown as mean ±SD.

Supplement 14

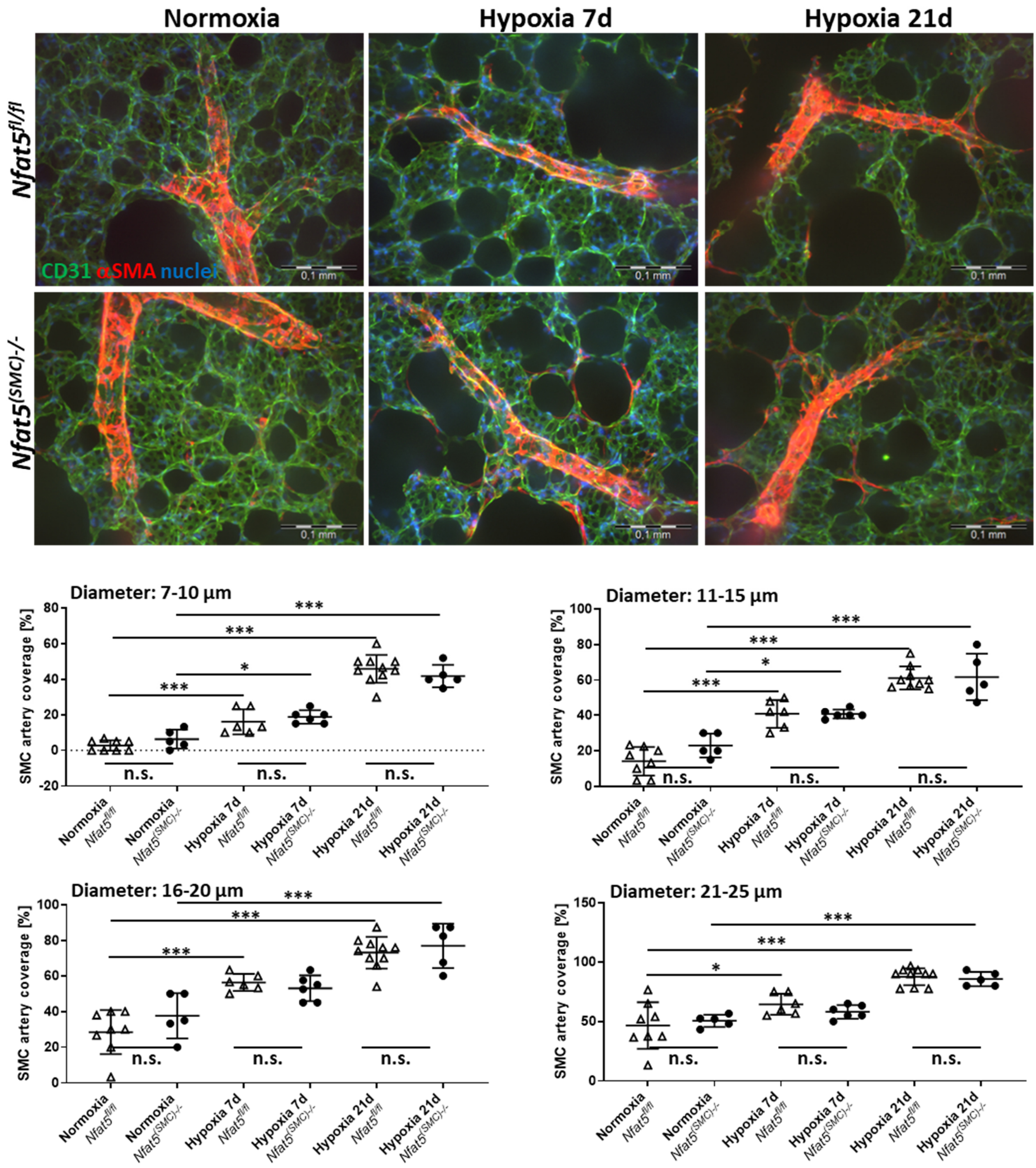

SMC coverage of arterial blood vessels with different caliber (extension of Figure 4). Vibratome sections of lungs from hypoxia/normoxia-exposed *Nfat5*$^{(SMC)-/-}$ and *Nfat5*$^{fl/fl}$ mice were processed to detect CD31 (green) and αSMA (red) by immunofluorescence-based techniques (scale bars: 100 µm). Image stacks of confocal images were morphometrically evaluated to determine the coverage of arterial segments with different caliber comprising the following groups: 7-10 µm, 11-15 µm, 16-20 µm and 21-25 µm (n.s. – not significant, *p<0.05, **p<0.01, ***p<0.001 as indicated, n=5-10).
